# Multi-applications of carbon dots and polydopamine-coated carbon dots for Fe^3+^ detection, bioimaging, dopamine assay and photothermal therapy

**DOI:** 10.1186/s11671-023-03809-5

**Published:** 2023-03-02

**Authors:** Jun Chen, Yuting Wang, Liang Wang, Mingjie Liu, Linlin Fang, Peng Chu, Chuanzhou Gao, Dapeng Chen, Dongze Ren, Jianbin Zhang

**Affiliations:** 1 Collage of Pharmacy, Dalian Medical University, 9 West Sect Lvshun South Rd, Dalian, 116044 China; 2 Laboratory Animal Center, Dalian Medical University, Dalian, 116044 China; 3 Institute of Cancer Stem Cell, Dalian Medical University, Dalian, 116044 China

**Keywords:** Carbon dots, Polydopamine, Fe^3+^ detection, Bioimaging, Photothermal therapy, Biomass

## Abstract

Carbon dots (CDs) or CDs/polymer composites have been applied in numerous fields. Here, novel CDs were synthesized by carbonization of egg yolk, and characterized by TEM, FTIR, XPS and photoluminescence spectra. The CDs were found to be approximate sphere in shape with an average size of 4.46 ± 1.17 nm, and emitted bright blue photoluminescence under UV irradiation. The photoluminescence of CDs was found selectively quenched by Fe^3+^ in a linear manner in the range of 0.05–0.45 mM, meaning they could be applied for Fe^3+^ detection in solution. Moreover, the CDs could be uptaken by HepG2 cells to exhibit bright blue photoluminescence. The intensity could reflect the level of intracellular Fe^3+^, indicating they could be further used for cell imaging and intracellular Fe^3+^ monitoring. Next, dopamine was polymerized on the surface of CDs to obtain the polydopamine (PDA)-coated CDs (CDs@PDA). We found PDA coating could quench the photoluminescence of CDs via inner filter effect, and the degree of quenching was linearly related to the logarithm of DA concentration (Log C_DA_). Also, the selectivity experiment indicated the method had a high selectivity for DA over a number of possible interfering species. This indicated the CDs in combination with Tris buffer could be potentially applied as the assay kit of dopamine. At last, the CDs@PDA exhibited excellent photothermal conversion capability, and they could efficiently kill HepG2 cells under NIR laser irradiation. Overall, the CDs and CDs@PDA in this work exhibited many excellent advantages, and could be potentially used for multi-applications, such as Fe^3+^ sensor in solution and cellular, cell imaging, dopamine assay kit, as well as photothermal agents for cancer therapy.

**Supplementary Information:**

The online version contains supplementary material available at 10.1186/s11671-023-03809-5.

## Introduction

Since firstly reported in 2004, carbon dots (CDs) have become one of the most representative materials with various unique inherent properties [1]. They are zero-dimensional carbon nanoparticles with particle sizes less than 10 nm, emitting bright photoluminescence under UV excitation. Generally, CDs can be easily prepared by “top-down” method (i.e., laser ablation, arc discharge and electrochemical methods) and “bottom-up” method (i.e., hydrothermal, microwave, thermal decomposition, carbonization, pyrolysis, solvothermal, ultrasonic methods) [2]. In comparison with traditional semiconductor quantum dots and organic dyes, CDs have a variety of advantages such as low toxicity, excellent water solubility, high photostability, adjustable photoluminescence, low cost and easy modification. These excellent properties endow CDs with multitudinous applications in optoelectronics, chemistry and biology, including photocatalysis, energy storage, optical device, sensors, bioimaging, drug delivery and fluorescent ink, etc. [3–6].

Ferric ion (Fe^3+^) is an essential trace element, extensively distributed in the human body. It is an important component of hemoglobin, myoglobin, cytochrome A and some respiratory enzymes, which plays an irreplaceable role in oxygen transport and metabolism [7, 8]. Fe^3+^ deficiency can cause some disorders such as anemia, Parkinson's disease, Alzheimer’s disease, and irreversibly affect children's intellectual development [9]. Furthermore, excess Fe^3+^ is toxic and leads to severe adverse effect such as liver and kidney damage (hemochromatosis), neurodegenerative, osteoporosis and cancers [10]. In addition, it is worth noting that Fe^3+^ is also one of the main causes of water pollution. Therefore, it is of great significance to detect Fe^3+^ in external environment and living organism for human health [11]. Recently, CDs are proven to be convenience, rapid and hypersensitive sensors of Fe^3+^. They have been widely applied for quantitatively detect the concentration of Fe^3+^ in pollutants, as well as qualitatively reflect the level of Fe^3+^ in cells by bioimaging. Chang et al*.* synthesized an environmentally friendly CDs and designed a rapid and label-free ‘‘turn-off” sensing platform for ultrasensitive recognition of Fe^3+^ in vitro and in vivo [12]. Liu et al*.* synthesized CDs by hydrothermal carbonization of *Fusobacterium nucleatum* (Fn-CDs). The fluorescent Fn-CDs were very sensitive to the presence of Fe^3+^ ions even in cells, exhibiting great promising applications in vivo detection of Fe^3+^ ions [13]. In our previous studies, we prepared two kinds of CDs using cherry blossom flowers and *Prunus cerasifera* fruits. Both CDs could be selectively quenched by Fe^3+^ and serve as the “turn-off” sensors [14, 15].

Apart from being used alone, CDs in combination with functional molecules/materials to produce novel composites has gained increasing interest of researchers. CDs/polymer composites, such as CDs/PVA, CDs/acrylamide, CDs/chitosan, and CDs/alginate, can effectively overcome some shortcomings of pure CDs and increase their multipurpose applications [16]. Polydopamine (PDA) is a promising melanin-like material, obtained from the oxidation and self-polymerization of dopamine (DA) [17]. PDA has many excellent properties such as good biocompatibility, degradability, antioxidant activity, high photothermal conversion efficiency and strong metal ion chelation [18]. Generally, CDs in combination with PDA nanoparticles could bring them some new functions and enrich the scope of application. For example, they could be developed for photothermal therapy, as PDA can efficiently convert near-infrared (NIR) light into thermal energy in malignant lesions to induce cancer cell apoptosis or necrosis [19]. Zhang et al. developed the multifunctional Mn^2+^ complex-modified PDA and CDs-based nanoparticles (PDA@N-CDs(Mn) NPs), and successfully used them for trimodality fluorescent, photothermal and magnetic resonance (MR) imaging in vitro and in vivo [20]. Besides in form of nanoparticles, coating is another useful application of PDA. It has been widely coated on the surface of metals, oxides, ceramics, polymer, micro and nanoparticles [21]. Evidently, CDs can also be coated by PDA (named CDs@PDA) for numerous applications [22]. For instance, some researchers found the PDA coating could influence the photoluminescence intensity of CDs, and successfully used them to detect of 4-nitrophenol, Fe^3+^, glutathione and dopamine [23–25]. Interestingly, it seems the coating of PDA may cause different effects on the photoluminescence properties of CDs. Chaiendoo et al*.* found the polymerization of DA on the surface could enhance the photoluminescence intensity of CDs with a linear relationship [24]. However, Zhu et al. reported that PDA coating could quench the photoluminescence of graphene quantum dots via fluorescence resonance energy transfer (FRET) [25]. Despite the promising potentials of CDs, the reports of PDA-coated CDs were still very few.

**Scheme 1:**
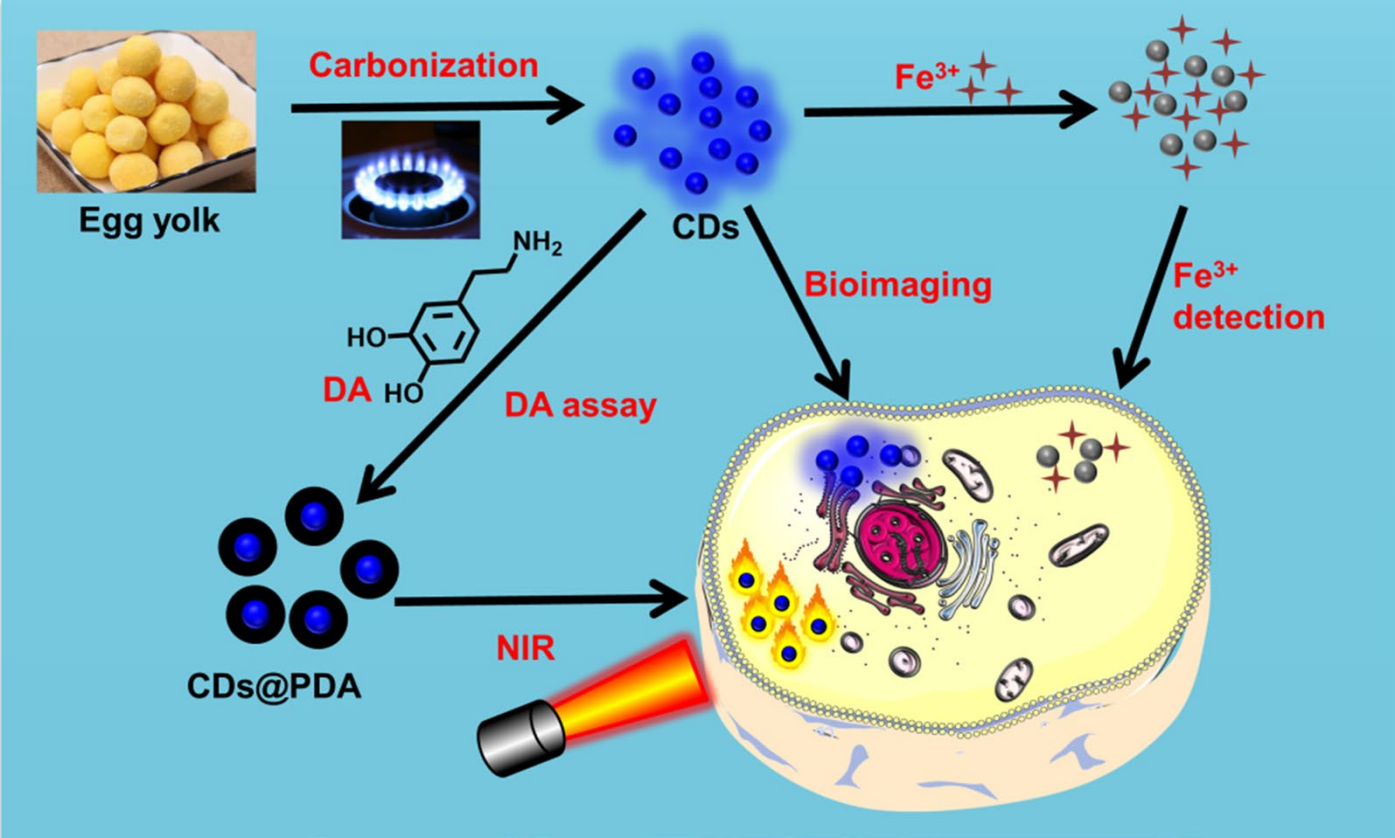
Schematic illustration of the synthesis of CDs and CDs@PDA, as well as their potential applications in sensors, bioimaging and photothermal therapy

Due to the excellent advantages of CDs and PDA, in this work, we aimed to develop novel CDs and coat them with PDA to further explore their potential applications. As seen in Scheme 1, CDs were firstly synthesized by carbonizing egg yolk on gas flame and characterized. Subsequently, the response of CDs to metal ions was evaluated; founding the photoluminescence could be selectively quenched by Fe^3+^ in a linear manner. Furthermore, the cytotoxicity and cell imaging of CDs were carried out, which showed that CDs were harmless and exhibited bright blue photoluminescence in HepG2 cells. Importantly, the photoluminescence intensity could also reflect the concentration of intracellular Fe^3+^. Next, CDs@PDA were prepared by polymerizing different amounts of DA on the surface of CDs. We found the photoluminescence of CDs could be quenched by PDA, which could be applied for monitoring the concentration of DA. The photothermal effect of CDs@PDA was finally studied, founding they significantly increase the temperature and kill cancer cells under NIR laser irradiation.

## Materials and methods

### Materials

Chicken eggs were bought from the local market. K_2_SO_4_, AgNO_3_, SnCl_2_·4H_2_O, BaCl_2_, Pb(NO_3_)_2_, CuSO_4_·5H_2_O, MgSO_4_·7H_2_O, MnSO_4_·H_2_O, FeCl_2_·4H_2_O, FeCl_3_, ZnCl_2_, NaCl,CaCl_2_, L-alanine, L-lysine, L-phenylalanine, L-cysteine, L-arginine, L-glycine, L-aspartic acid, L-glutamine, glucose, fructose, urea and glutathione (GSH) were purchased from Adamas-Beta Co., Ltd. (Shanghai, China). Dopamine hydrochloride and tris(hydroxymethyl)aminomethane (Tris) were obtained from 9 Ding Chemistry Co., Ltd. (Shanghai, China). 96-well plates (In vitro scientific) were obtained from Xinyou Biotechnology Co., Ltd. (Hangzhou, China). 6-well plates were obtained from Nest Biotechnology Co., Ltd. (Wuxi, China). Black opaque 96-well plates were supported by Jet Bio-Filtration Co., Ltd. (Guangzhou, China). Dulbecco’s modified Eagle’s medium (DMEM), trypsin, penicillin, streptomycin, 3-(4,5-dimethylthiazol-2-yl)-2,5-diphenyltetrazolium bromide (MTT), propidium iodide (PI) and Calcein-AM were bought from Solarbio Science & Technology Co., Ltd. (Beijing, China). Fetal bovine serum (FBS) was purchased from Zhejiang Tianhang Sijiqing Biotechnology Co., Ltd., (Hangzhou, China). All of the chemicals were analytical grade and used without further purification.

### Synthesis and characterizations of CDs

CDs were synthesized by the carbonization of egg yolk with some modifications [26]. Briefly, five cooked egg yolks were separated from hard-boiled chicken eggs. They were subsequently put in a frying pan over the gas flame and carbonized for 15 min. After cooled to room temperature, the residue was collected and dispersed into Milli-Q deionized water. The mixture was sonicated for 8 h by an ultrasonic cleaner, and then centrifuged for 30 min at the speed of 10,000 rpm. The supernatant was collected and filtered by 0.22-μm membrane filter to remove large particles. Finally, the sample was lyophilized by a freeze-dryer (SCIENTZ-10N, Ningbo Scientz Biotechnology Co., Ltd., Zhejiang, China) to obtain CDs.

The photoluminescence spectra and ultraviolet–visible (UV–Vis) absorption spectrum were measured by a Synergy H1 Hybrid Multi-Mode Microplate Reader (BioTek, Winooski, VT, USA). Transmission electron microscopic (TEM) images were acquired on a JEM-2100 microscope (JEOL, Japan). Fourier transform infrared spectroscopic (FTIR) spectra were recorded on an IRPrestige-2 FTIR spectrophotometer (Shimadzu, Japan). X-ray photoelectron spectroscopic (XPS) measurement was carried out on an ESCALAB 250Xi spectrometer (Thermo, USA). The lifetime measurements were performed on a Fluoromax-4 spectrofluorometer (Horiba, UK).

CDs aqueous dispersions with pH in the range of 1–14 were prepared using a pH–Stat automatic titration unit (848 Titrino plus, Metrohm AG, Herisau, Switzerland), and the photoluminescence intensities were recorded (*λ*_ex_ = 360 nm, *λ*_em_ = 443 nm).

### Quantum yield measurement

The quantum yield (QY) of CDs was measured according to the previously reported method [27], which used quinine sulfate in 0.1 M H_2_SO_4_ as the reference (QY = 0.54). Briefly, certain amounts of CDs and quinine sulfate were, respectively, dissolved in water and 0.1 M H_2_SO_4_. The absorbance values at 360 nm were measured, which should be kept under 0.05. Furthermore, the integral photoluminescence intensities of CDs and quinine sulfate were recorded within the emission wavelength of 405–600 nm, excited at the wavelength of 360 nm. The QY was calculated by according to the following equation:

1 $${\text{QY}} = {\text{QY}}_{{{\text{Ref}}}} \times \frac{I}{{I_{{{\text{Ref}}}} }} \times \frac{{A_{{{\text{Ref}}}} }}{A} \times \frac{{\eta^{2} }}{{\eta_{{{\text{Ref}}}}^{2} }}$$

where QY is the quantum yield, *I* is the measured integral photoluminescence intensity, *A* refers to the absorbance. In addition, *η* presents the refractive index of the solvent, which were 1.33 for both CDs aqueous dispersion and quinine sulfate solution. The subscript *Ref* refers to the corresponding parameter of quinine sulfate.

### Detection of Fe^3+^ in solution

The response of CDs to metal ions was operated according to previously reported method [15]. Briefly, 150 μL CDs aqueous dispersion was, respectively, mixed with 150 μL inorganic salt solution containing 2 mM of K_2_SO_4_, AgNO_3_, SnCl_2_·4H_2_O, BaCl_2_, Pb(NO_3_)_2_, CuSO_4_·5H_2_O, MgSO_4_·7H_2_O, MnSO_4_·H_2_O, FeCl_2_·4H_2_O, FeCl_3_, ZnCl_2_, NaCl and CaCl_2_. Then, 200 μL of each mixture was transferred into a black opaque 96-well plate (Jet Bio-Filtration, Guangzhou, China) and measured by a BioTek microplate reader (*λ*_ex_ = 360 nm, *λ*_em_ = 443 nm).

To quantitatively determination of Fe^3+^, CDs were mixed with various concentrations of Fe^3+^ (0–1 mM) and the photoluminescence intensities were recorded with the same experimental conditions as described above. All experiments were conducted at room temperature.

### Cytotoxicity assay of CDs

HepG2 cells were cultured in DMEM, containing 10% fetal bovine serum (FBS), 1% penicillin and 1% streptomycin at 37 ℃. MTT assay was adopted to evaluate the cytotoxicity of CDs. Firstly, HepG2 cells were seeded in a 96-well plate with the density of 1 × 10^4^ cells/well and cultured for 24 h in under 5% CO_2_ at 37 ℃. Then, the medium was replaced by 100 µL DMEM medium containing CDs of different concentrations (0, 3.9, 7.8, 15.6, 31.2, 62.5, 125, 250, 500 and 1000 µg/mL) and incubated for another 24 h. After that, the medium was discarded and equal volume of DMEM with 5 mg/mL MTT was added in each well. After 4 h incubation, the supernatant was discarded and 200 μL DMSO was added to each well to dissolve the formazan crystals. Finally, the absorbance was measured at 570 nm with a microplate reader.

### Cell imaging and intracellular Fe^3+^ detection

HepG2 cells in the logarithmic growth phase were seeded in a 6-well glass bottom plate (2 × 10^5^ cells/well) and cultured for 24 h. Then, the supernatant was removed and washed three times with PBS. Fresh DMEM with 1 mg/ml CDs was added and incubated for another 24 h. After that, the supernatant was removed and washed three times with PBS. The medium with various concentrations of Fe^3+^ (0, 200, 500 and 1000 µM) was added and further incubated for 2 h. At last, the cells were washed for three times and observed by an inverted photoluminescence microscope (IX81, Olympus, Tokyo, Japan).

### Synthesis and characterizations of CDs@PDA

CDs@PDA were prepared by polymerizing different amounts of DA on the surface of CDs. Briefly, CDs were dissolved in Tris buffer (10 mM, pH = 8.5) with the concentration of 666.7 µg/mL. Then, different amounts of dopamine hydrochloride were added into CDs aqueous dispersion forming the concentrations of 2.6, 5.2, 10.4, 20.8, 41.6, 83.3, 166.7, 333.3 µg/mL. The mixtures were shaken for 12 h at 37 ℃, and then centrifuged for 30 min at the speed of 10,000 rpm. The supernatant was collected and filtered by 0.22-μm membrane filter to remove large particles. Finally, the CDs@PDA were lyophilized by a freeze-dryer, with CDs/DA ratio of 256:1, 128:1, 64:1, 32:1, 16:1, 8:1, 4:1 and 2:1 (w/w).

The characterizations of CDs@PDA were also conducted with the same method of CDs.

### Dopamine assay

CDs were dissolved in Tris buffer (10 mM, pH = 8.5) with the concentration of 666.7 µg/mL. Then, different amounts of dopamine hydrochloride were added into CDs aqueous dispersion forming the concentrations of 2.6, 5.2, 10.4, 20.8, 41.6, 83.3, 166.7, 333.3 µg/mL. The mixtures were shaken for 12 h at 37 ℃, the photoluminescence intensities were recorded by a BioTek microplate reader (*λ*_ex_ = 360 nm, *λ*_em_ = 443 nm).

To investigate the effect of other substances on the sensitivity of DA assay, 166.7 µg/mL of DA was mixed with 100 µM of interferences. Then they were operated in the same way as described above. The interferences included K_2_SO_4_, AgNO_3_, BaCl_2_, Pb(NO_3_)_2_, CuSO_4_·5H_2_O, MnSO_4_·H_2_O, ZnCl_2_, NaCl, CaCl_2_, L-alanine, L-lysine, L-phenylalanine, L-cysteine, L-arginine, L-glycine, L-aspartic acid, L-glutamine, glucose, fructose, urea and glutathione (GSH).

### NIR triggered photothermal performance

To confirm the photothermal effect performance, 1 mL CDs@PDA aqueous dispersion with different CDs/DA ratios was added in a quartz cuvette, maintaining the concentration of CDs at 666.7 µg/mL. Then, the aqueous dispersions were irradiated by an 808 nm NIR laser at 1 W/cm^2^ for 10 min, and the temperature was recorded. In addition, the thermal imaging of each sample was recorded by an IR thermal camera.

### Cellular photothermal effect

The photothermal therapeutic effect of CDs@PDA on cellular level was evaluated by MTT assay and live-dead staining. Similar to the cytotoxicity of CDs, HepG2 cells were seeded in a 96-well plate and cultured for 24 h. And then, the cells were incubated with different types of CDs@PDA, which contained 666.7 µg/mL CDs. After 4 h incubation, the cells were irradiated by NIR laser at 2 W/cm^2^ for 3 min and cultured for another 12 h. Finally, MTT was added to detect the cell viabilities.

For in vitro live-dead staining, HepG2 cells were seeded in a 6-well plate and cultured for 24 h. Then, DMEM containing different types of CDs@PDA was added and incubated for another 4 h. The cells were subsequently irradiated by NIR laser at 2 W/cm^2^ for 3 min and cultured for another 12 h. At last, the cells were washed and stained with Calcein-AM and PI, and immediately observed by an Olympus IX81 photoluminescence microscope.

## Results and discussions

### Characterizations of CDs

**Fig. 1:**
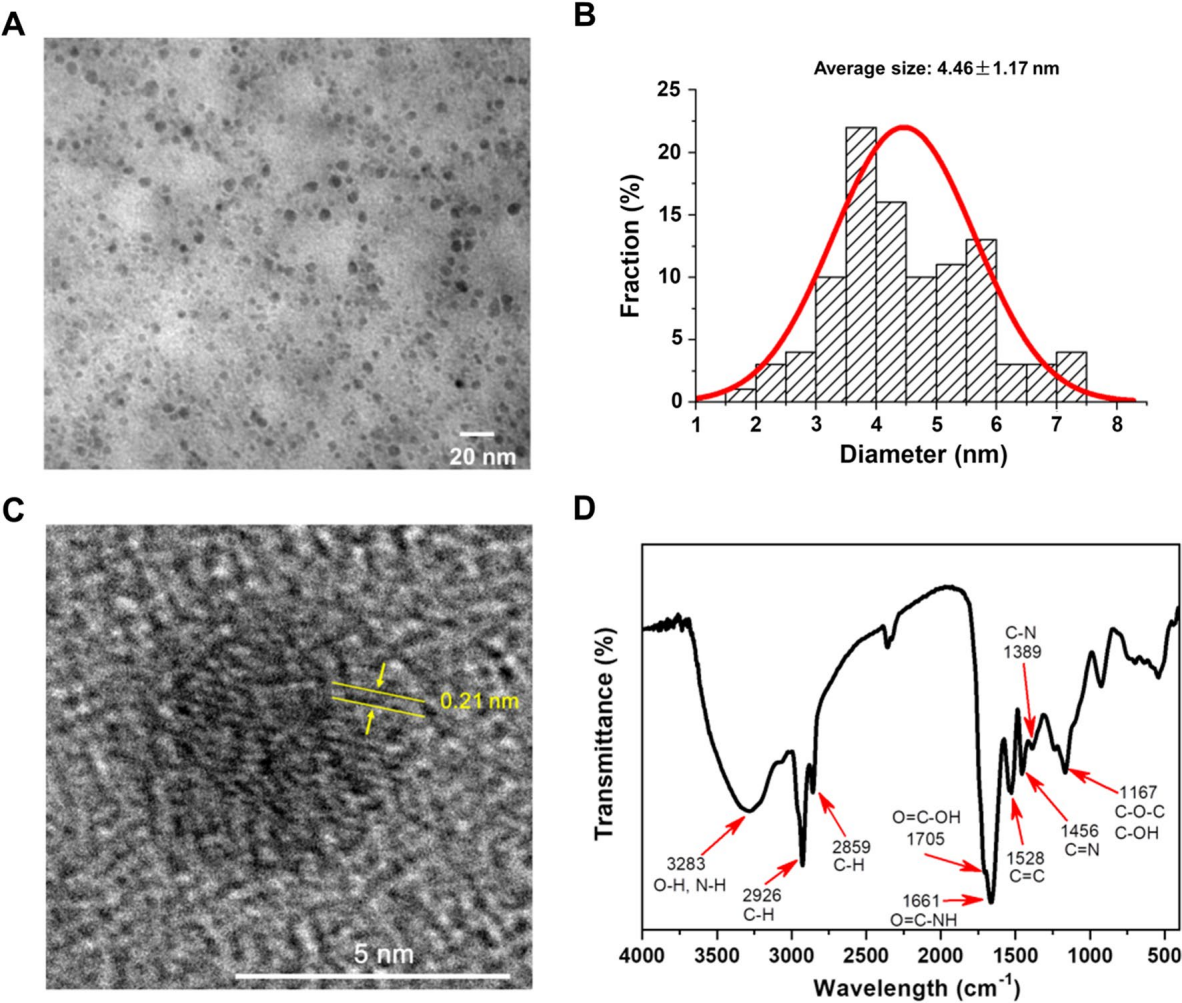
**A** TEM image, **B** size distribution, **C** HRTEM image and **D** FTIR spectrum of CDs

As illustrated in Scheme 1, the CDs were directly synthesized through a carbonization process on gas flame using egg yolk as carbon source. In comparison with commonly reported hydrothermal method and laser ablation, the advantages of this method are convenient operation, short time, low cost, eco-friendly and easily mass production. The charred egg yolk was dark powder, and CDs could be separated from it by sonication treatment. The obtained CDs were pale brown, which had very bright blue photoluminescence in water. The characterizations of CDs were, respectively, described by TEM images, as well as spectra of FTIR and XPS. Figure 1A showed TEM image of CDs, showing they were approximate sphere in shape with good monodispersity. The sizes of CDs were uniform and mainly distributed in the range of 3–6 nm. The average diameter of randomly selected one hundred CDs was calculated to be 4.46 ± 1.17 nm (Fig. 1B). Moreover, the HRTEM image (Fig. 1C) clearly exhibited the lattice fringes with a spacing of 0.21 nm, which was attributed to the (100) facet of graphite.

**Fig. 2:**
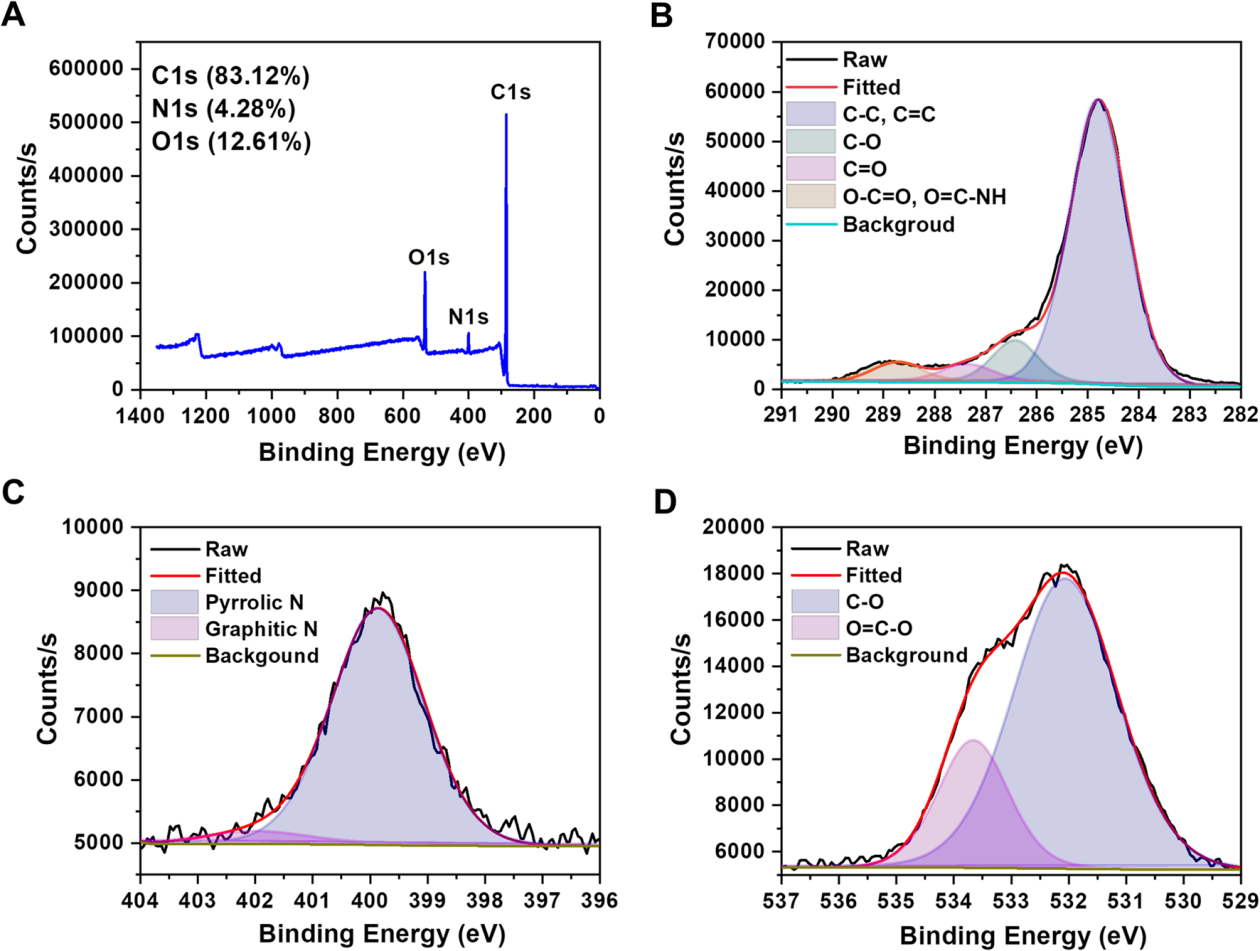
**A** XPS survey spectrum of CDs. The high resolution XPS C1s (**B**), N1s (**C**) and O1s (**D**) spectra of CDs

The functional groups on the surface of CDs were measured by FTIR spectroscopy. As shown in Fig. 1D, the absorption band at 3283 cm^−1^ was attributed to the stretching vibrations of O–H and N–H. The peaks at 2926 cm^−1^ and 2958 cm^−1^ were assigned to the stretching vibrations of C–H. Furthermore, the peaks at 1705 cm^−1^ and 1661 cm^−1^ were originated from the C=O stretching vibrations of carboxy group (O=C–OH) and amide group (O=C–NH). The peaks at 1528, 1456 and 1389 cm^−1^ were caused by the stretching vibrations of C=C, C=N and C–N, respectively. The band at 1167 cm^−1^ indicated the present of ether linkage (C–O–C) or alcohol group (C–OH). XPS analysis was also conducted to further identify the chemical composition and structure of CDs. As seen in the full XPS of CDs (Fig. 2A), there main peaks at 288.1, 400.1 and 533.1 eV could be observed, corresponding to C1s, N1s and O1s. The percentages of C, N and O elements in CDs were 83.12%, 4.28% and 12.61%, respectively. High resolution XPS of each element was further performed and fitted to confirm the structure. The high resolution C1s spectrum in Fig. 2B could be deconvoluted into four main peaks at 284.8 eV, 286.4 eV, 287.3 eV and 288.7 eV, indicating the present of C–C/C=C, C–O, C=O and O–C=O/O=C–NH bonds. The N1s spectrum (Fig. 2C) could be divided into two peaks, with the pyrrolic N at 399.9 eV and the graphitic N at 402.0 eV. Similarly, the peaks of C-O (532.0 eV) and O=C–O (533.6 eV) bonds could be found in the O1s spectrum (Fig. 2D). The results of XPS were exactly consistent with that of FTIR. They manifested that many hydrophilic groups (such as hydroxyl, carboxyl and amino groups) were present on the surface of CDs, endowing the excellent aqueous solubility and enhanced photoluminescence property [28].

### Photoluminescence properties of CDs

**Fig. 3:**
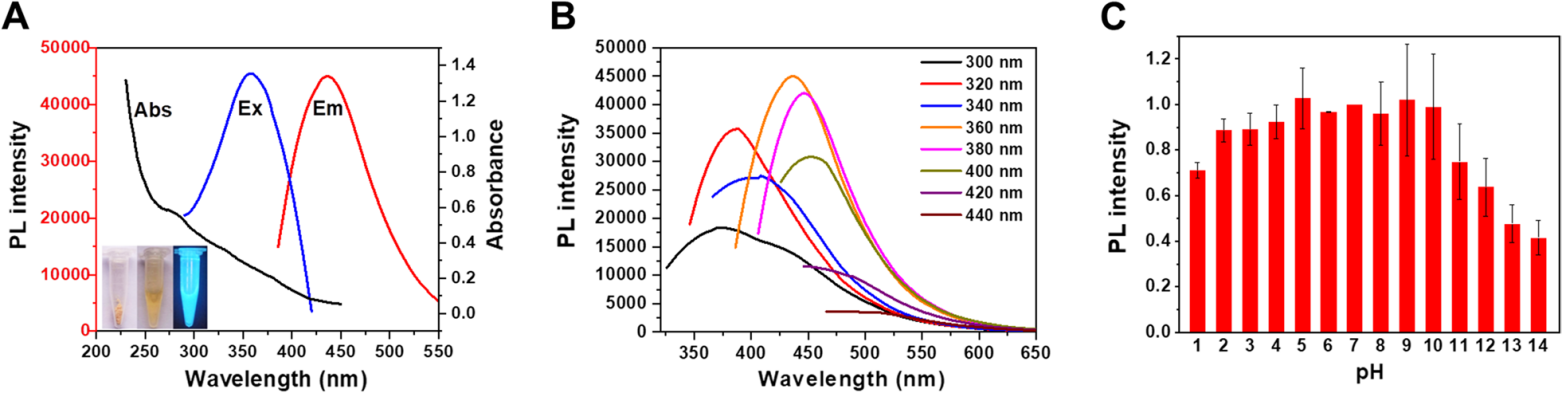
**A** UV–vis absorption (black line), photoluminescence excitation (Ex, blue line) and emission (Em, red line) spectra of CDs. **B** Photoluminescence emission spectra of CDs with different excitation wavelength. **C** Photoluminescence intensity of CDs at different pH condition

The photoluminescence properties CDs were crucial factors to determine the scope of application. Hence, their optical properties were firstly measured by UV–vis absorption and photoluminescence spectra. As seen in Fig. 3A, there is a weak absorption peak at 280 nm in the UV–vis absorption spectrum, which was ascribed to the n-π^*^ transition of the C=C [29]. In addition, the photoluminescence spectra of CDs exhibited the maximum excitation wavelength at 360 nm and the maximum emission wavelength of CDs 443 nm, with a general stoke shift of 83 nm. The insert showed the pale brown color of CDs powder and aqueous dispersion under daylight, with bright blue photoluminescence under UV lamp. The photoluminescence emission spectra of CDs with different excitation wavelength (300–440 nm) were also performed. As shown in Fig. 3B, the CDs exhibited excitation-dependent photoluminescence behavior. It seemed the emission peak shifted to longer wavelength with the increase of excitation wavelength. The intensity of CDs was generally increased by exciting from 300 to 360 nm, while it was gradually decreased from 360 to 440 nm. This variation in emission intensity with excitation wavelength could be attributed to the different energy levels incorporated into the CDs, by different surface groups such as C–O, C=O, C=N, C–N, C=C and O=C–NH [30]. Furthermore, the quantum yield (QY) of CDs was calculated to be 20.2% using quinine sulfate as the standard. The QY was significantly higher than that of various biomass-based CDs, which were generally less than 10%. The pH stability of CDs was also evaluated by comparing the relative photoluminescence intensity under different pH condition (*λ*_ex_ = 360 nm, *λ*_em_ = 443 nm). The intensity of pH 7 was set as the reference, and we found they were stable in the range of pH 3–10 with the intensity variation less than 10% (Fig. 3C). However, once the pH was below 1 or over 10, there was a significant decline of photoluminescence intensity. It was speculated that CDs might aggregate or be destroyed at strong acid or basic condition, and resulting the decline of photoluminescence intensity [31]. Fluorescent ink is invisible to the naked eye, and only in ultraviolet or infrared light luminescence.

### Sensitivity and detection of Fe^3+^

**Fig. 4:**
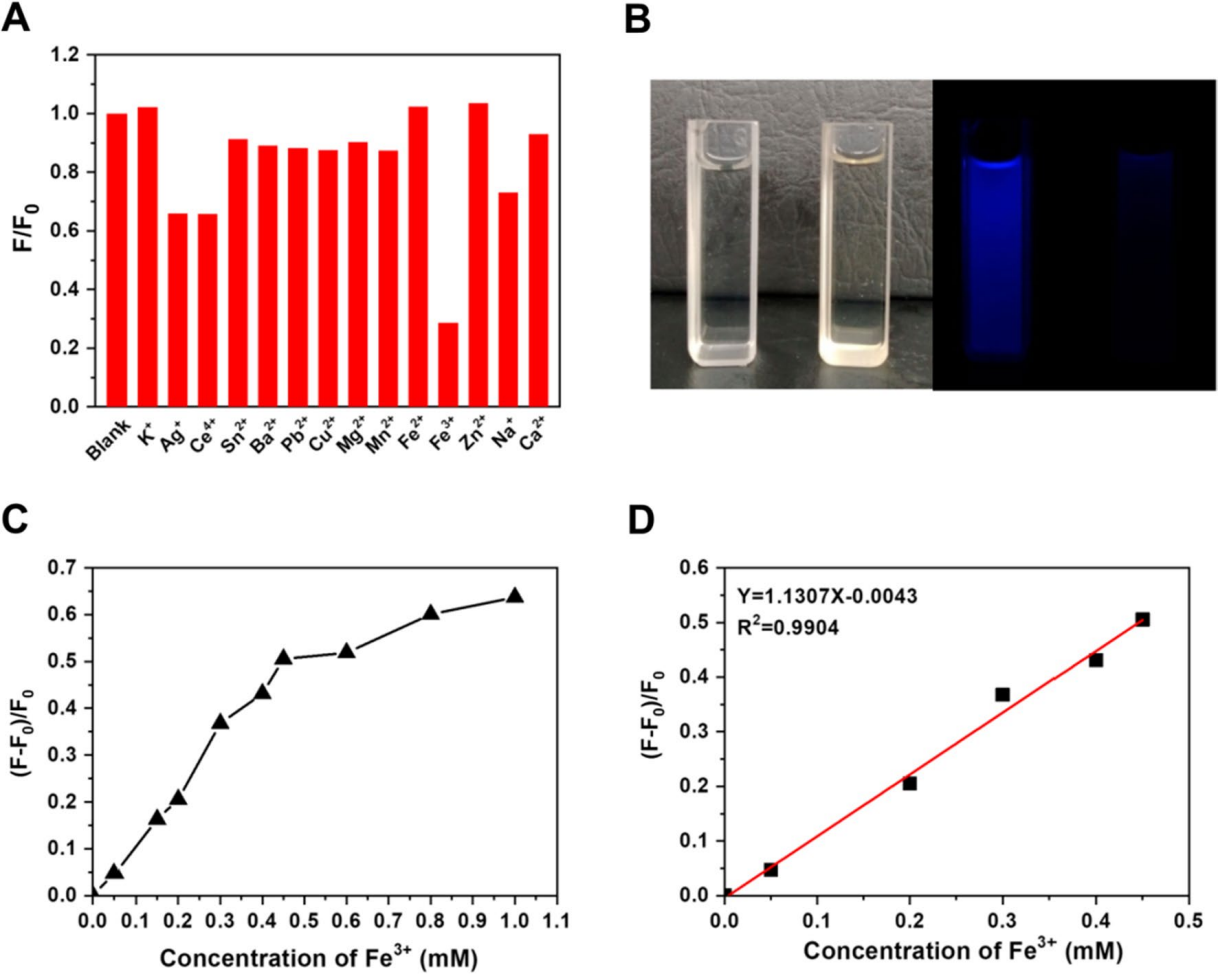
**A** Selectivity of CDs on Fe^3+^ over other metal ions, F_0_ and F present the photoluminescence intensity of CDs before and after adding metal ions. **B** Digital photographs of CDs with or without Fe^3+^ under daylight and UV light. **C** The relationship and **D** the linear curve of (F_0_ − F)/F_0_ versus the Fe^3+^ concentration

CDs could serve as sensors of lots of materials, such as metal ions, drugs, small molecule compounds and pesticides. Up to now, abundant researches have been proved that the photoluminescence properties of CDs can be applied into selective detection of metal ions depending on change of photoluminescence signal, including Fe^3+^, Ag^+^, Zn^2+^, Hg^2+^ and Cu^2+^ [32–34]. Especially, Fe^3+^ has been proven able to quench various types of CDs. In this work, the selective response of CDs toward multifarious metallic ions has been performed, by incubation CDs with 1 mM of K^+^, Ag^+^, Ce^4+^, Sn^2+^, Ba^2+^, Pb^2+^, Cu^2+^, Mg^2+^, Mn^2+^, Fe^2+^, Fe^3+^, Zn^2+^, Na^+^ or Ca^2+^. The photoluminescence intensities of CDs incubated with different metal ions (F) were compared with that of blank CDs (F_0_). The F/F_0_ value could reflect the extent of CDs response (enhance or quench) to each metal ion. As shown in Fig. 4A, most of the metal ions had insignificant effect on the photoluminescence intensity of CDs because the F/F_0_ values were close to 1, except Ag^+^, Ce^4+^, Fe^3+^ and Na^+^. It was obvious that CDs were most sensitive to Fe^3+^, with the F/F_0_ value decrease to 0.3. Furthermore, the blue photoluminescence of CDs was disappeared under UV irradiation, once Fe^3+^ was added (Fig. 4B). The high selectivity to Fe^3+^ might be attributed to the faster chelating process of Fe^3+^ toward N and O element of CDs than other metal ions. It was reported that the mechanism of quenching was caused by the electron transfer between CDs and Fe^3+^ [35]. Next, the relationship between CDs quenching and Fe^3+^ concentration was conducted (Fig. 4C and D). The degree of quenched CDs was expressed as (F_0_ − F)/F_0_, and it was gradually increased with the increase of Fe^3+^ concentration. There was a good linear relationship existed between (F_0_ − F)/F_0_ and Fe^3+^ concentration the range of 0.05–0.45 mM, which was fitted as (F_0_ − F)/F_0_ = 1.1307C(Fe^3+^) − 0.0043 (*R*^2^ = 0.9904). The limit of detection (LOD) was calculated to be 1.02 μM, according to the equation of LOD = 3*δ*/S. Where *δ* was calculated as the standard deviation of 11 blank CDs samples without Fe^3+^, and S was the slope (1.1307). The LOD was lower than the iron concentrations of the standard in drinking water (5.4 μM, regulated by the WHO) and blood (20–29 μM). In comparison with similar biomass-based CDs, the detection range of CDs in this work was significantly wider than that synthesized from Dwarf banana peel, Poa Pratensis and Betel leaves [36–38]. Hence, the CDs could be developed as specific sensor of Fe^3+^, and potentially applied for water pollution detection such as urban sewage and industrial waste. However, it worth noting that the Fe^3+^ detection might be affected by the present the interference factors, such as Ag^+^, Ce^4+^, and Na^+^.

### Cell imaging and intracellular Fe^3+^ detection

**Fig. 5:**
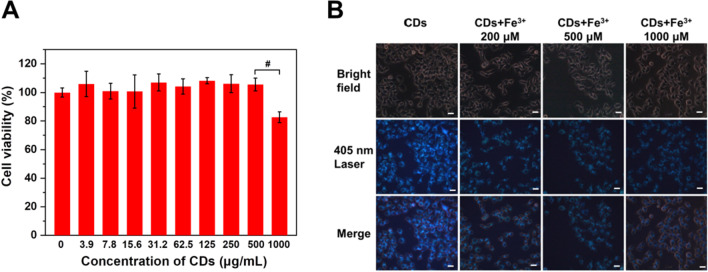
**A** Cellular viabilities of HepG2 cells in the present of different concentrations of CDs, ^#^*p* < 0.001. **B** Photoluminescence images of HepG2 cells incubated with CDs in the present of different Fe^3+^ (0–1 mM)

Due to the excellent biocompatibility and photoluminescence property, CDs could also be easily internalized by cells for bioimaging, and further reflect the cellular level of certain substance [39]. In this work, we studied the capabilities of CDs in bioimaging and intracellular Fe^3+^ detection. The cytotoxicity of CDs was firstly evaluated by MTT method with the result listed in Fig. 5A. We found the viabilities of HepG2 cells were almost 100%, when treated by CDs less than 500 μg/mL. In case the CDs concentration reached to 1000 μg/mL, the cell viability still maintained over 80%. Hence, the biocompatibility of CDs from egg yolk was excellent, which ensured their further biological applications. Figure 5B exhibited the image of HepG2 cells incubated with CDs. Bright blue photoluminescence was observed under 405 nm laser, demonstrating CDs had successfully entered into HepG2 cells. The photoluminescence intensity was strong in the area of cytoplasm and cell membrane, indicating CDs mainly distributed cytoplasm without entry into the nucleus. Furthermore, to explore the capability of intracellular Fe^3+^ detection CDs, HepG2 cells were incubated with different concentration of Fe^3+^ followed by CDs treatment. As seen in Fig. 5B, in comparison with untreated cells, the photoluminescence intensities of cells were significantly decreased after treated by 200, 500 and 1000 μM Fe^3+^.The decrease of photoluminescence intensity could be attributed to the internalization of Fe^3+^ by HepG2 cells and the following quench effect occurred. Moreover, we found the intracellular photoluminescence intensity was gradually decreased as the increase of Fe^3+^ concentration. This was because larger amount of Fe^3+^ was penetrated into cells if treated with higher concentration of Fe^3+^. In this work, the CDs could reflect the intracellular Fe^3+^ level in the micromolar range, which was the physiological intracellular amount. They might be potentially applied to detect the diseases associated with abnormal iron. Hence, the CDs could not only being applicable for cell imaging, but also being able to sever as intracellular Fe^3+^ monitor, as the photoluminescence signals could reflect the level of intracellular Fe^3+^.

### Characterizations of CDs@PDA

**Fig. 6:**
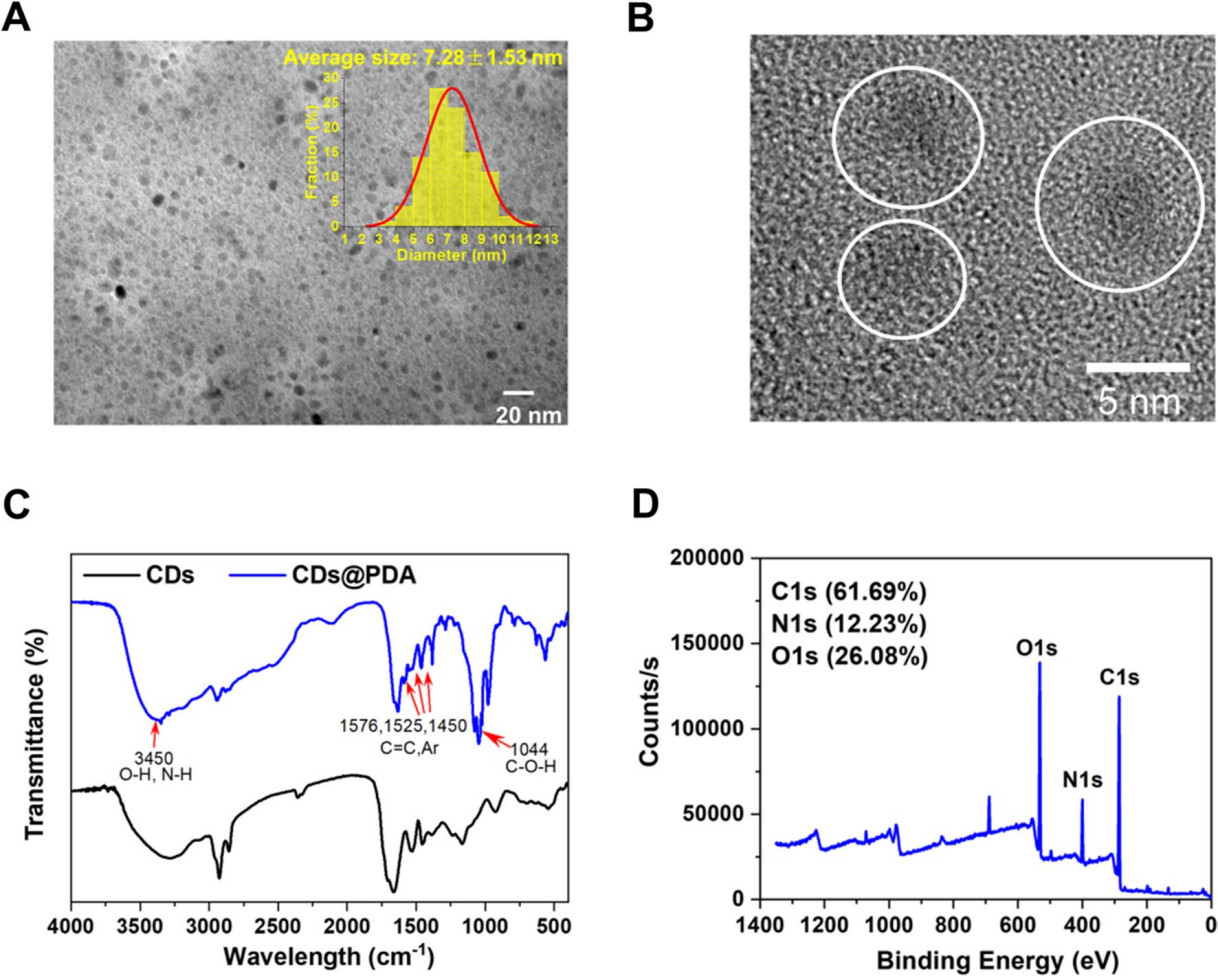
**A** TEM image, **B** HRTEM image, **C** FTIR spectrum and **D** XPS survey spectrum of CDs@PDA, with CDs/DA ratios of 8:1

PDA-coated CDs (CDs@PDA) were prepared by the polymerization of DA in the condition of basic Tris buffer. The characterizations of CDs@PDA were also described by TEM images, FTIR spectrum and XPS spectra. As exhibited in the TEM image of Fig. 6A, the morphology of CDs@PDA was similar to that of CDs, with nearly sphere in shape and good uniform dispersion. The average diameter of CDs@PDA was measured to be 7.28 ± 1.53 nm, slightly larger than CDs (4.46 ± 1.17 nm). In the HRTEM image (Fig. 6B), we found the structure of CDs@PDA contained two parts: the inner CDs core with obvious crystal structures and the outer PDA shell (the edge showed by white line). Furthermore, FTIR spectrum of CDs@PDA was compared with that of CDs. As polydopamine contains numerous hydroxyl and aryl, their characteristic peaks could be observed in the spectrum of CDs@PDA (Fig. 6C). The peaks at 3450 cm^−1^ was assigned to the stretching vibrations of O–H and N–H. The absorption band at 1450 cm^−1^, 1525 cm^−1^ and 1576 cm^−1^ were attributed to the stretching vibrations of C=C and aryl. The band at 1044 cm^−1^ indicated the present of C–OH. The chemical composition and structure of CDs@PDA were also analyzed by XPS spectra. As seen in Fig. 6D, the XPS survey spectrum of CDs@PDA had three main peaks at 286.1, 400.2 and 532.5 eV, corresponding to C1s, N1s and O1s. It worth noting that, the percentages of N and O elements have increased to 13.73% and 21.75% in comparison with CDs. This was because PDA contained abundant hydroxyl and amino groups. The high solution C1s, N1s and O1s XPS spectra of CDs@PDA were deconvoluted, and the peaks were similar to that of CDs (Fig. S1). The results fully demonstrated that PDA had successfully coated on the surface of CDs.

### Dopamine detection

**Fig. 7:**
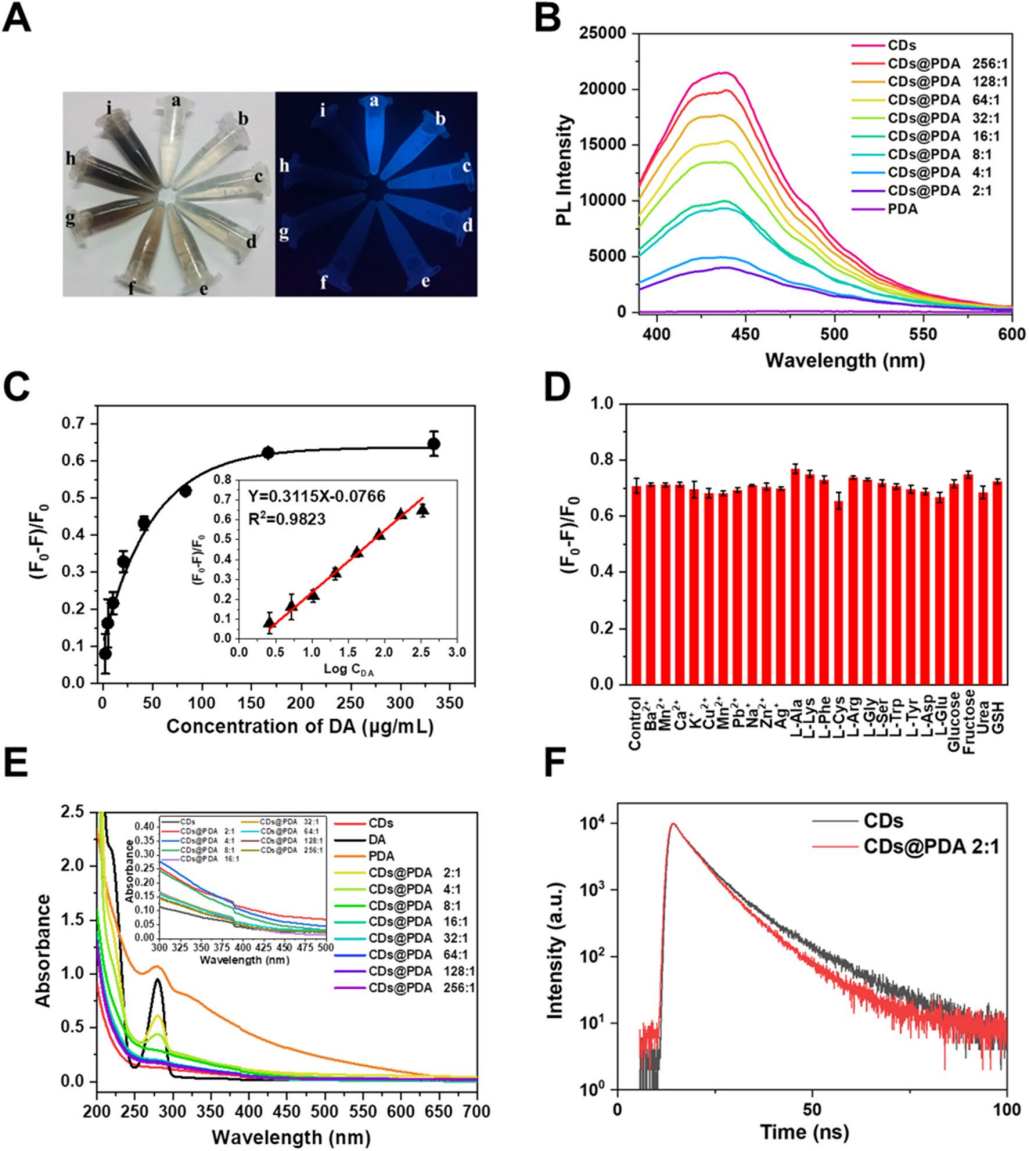
**A** Digital photographs of (a) CDs and (b–i) CDs@PDA under daylight or UV light, with CDs/DA ratios of (b) 256:1, (c) 128:1, (d) 64:1, (e) 32:1, (f) 16:1, (g) 8:1, (h) 4:1 and (i) 2:1. **B** Photoluminescence emission spectra of CDs@PDA with different CDs/DA ratios. **C** The relationship of (F_0_ − F)/F_0_ versus the DA concentration and inset shows the linear calibration curve of (F_0_ − F)/F_0_ versus the logarithm of DA concentration (Log C_DA_). **D** The effect of different interferences on the DA assay, with the concentrations of DA: 166.7 µg/mL, interferences: 100 µM. **E** UV–vis absorption spectra of CDs, DA and CDs@PDA with different CDs/DA ratios. Insert is the absorbance from 400 to 500 nm. **F** The lifetime decay curves of CDs and CDs@PDA, with CDs/DA ratio of 2:1

Furthermore, we prepared CDs@PDA with different CDs/DA ratios (256:1, 128:1, 64:1, 32:1, 16:1, 8:1, 4:1 and 2:1, w/w) to compare their photoluminescence properties. The appearance of CDs and CDs@PDA aqueous dispersions were observed under daylight or UV light (Fig. 7A). The concentration of CDs was maintained as 666.7 µg/mL, and the DA were gradually increased from 0 to 333.3 µg/mL. It was clear that color of CDs@PDA aqueous dispersion was gradually turned black as the increase of DA concentration, because PDA was structurally similar to naturally occurring melanin with black color. It seemed PDA had a quenching effect on the CDs, as the blue photoluminescence was gradually disappeared as the increase of PDA concentration under UV light. The exact effect of PDA coating on CDs was further conducted by comparing the photoluminescence intensities of various CDs@PDA (*λ*_ex_ = 360 nm, *λ*_ex_ = 443 nm). As seen in Fig. 7B, the intensities CDs@PDA were gradually decreased with the increased of PDA (CDs/DA ratios from 256:1 to 2:1), which was consistent with the observation under UV light. Subsequently, the degree of quenching (F_0_-F)/F_0_ was compared with the concentration of reacted DA, where the concentration of CDs was fixed as 666.7 µg/mL. It was quite clear that the degree of quenching (F_0_ − F)/F_0_ was gradually increased, with the concentration of reacted DA increased from 2.6 to 333.3 µg/mL (shown in Fig. 7C). As the curve of (F_0_ − F)/F_0_ versus DA concentration (C_DA_) seemed like logarithmic other than linear, the logarithmic plot of DA concentration (Log C_DA_) was further introduced. The inset exhibited a good linear correlation between (F_0_ − F)/F_0_ and Log C_DA_, with the fitted linear equation of (F_0_ − F)/F_0_ = 0.3115LogC_DA_ − 0.0766 (*R*^2^ = 0.9823). The LOD was calculated to be 3.70 μg/mL. This indicated that CDs were highly sensitive to the coating of PDA, and the change of photoluminescence of could exactly quantify the concentration of DA. As real samples contain many substances, which might interfere the accuracy of DA detection. To verify the selectivity and specificity of this method, various representative substances were mixed with DA including metal ions, amino acids and biomolecules. As shown in Fig. 7D, all the interferences have no obvious effect on the degree of quenching (F_0_ − F)/F_0_. Hence, the CDs in combination with Tris buffer could be potentially applied as the assay kit of dopamine with high selectivity and specificity. As far as we known, this is the first report of dopamine detection by CDs based on the polymerization reaction to form CD-coated PDA.

Generally, there are some types of quenching mechanisms, such as dynamic quenching, static quenching, Förster resonance energy transfer (FRET), photoinduced electron transfer (PET), surface energy transfer (SET), Dexter energy transfer (DET) and inner filter effect (IFE) [23]. To understand the quenching mechanism of PDA on CDs, we measured their UV–vis absorption spectra, emission spectra and lifetime decay curves. PDA had negligible photoluminescence emission (Fig. 7B), which was not overlapped with the emission spectrum of CDs, hence FRET could not occur. PDA had a broad-band absorption ranging from 200 to 600 nm, with the peak at 280 nm (Fig. 7E). It could enhance the absorbance of CDs@PDA from 360 to 443 nm, as the increase of PDA amounts. As the absorption of PDA was overlapped with the excitation and emission spectra of CDs, IFE might occurred during CDs quenching. However, the lifetime decay curve of CDs had been changed by PDA, when it coated on the surface (Fig. 7F). The lifetime of CDs was calculated to be 7.96 ns, and it was significantly decreased after coated by PDA (Fig. S2). All of the lifetimes of CDs@PDA were less than 7.0 ns, which even decreased to 6.73 nm for CDs@PDA with CDs/DA ratio of 2:1. The alteration of lifetime demonstrated the quenching mechanism was dynamic quenching, rather than IFE or static quenching.

### Photothermal effect of CDs@PDA

**Fig. 8:**
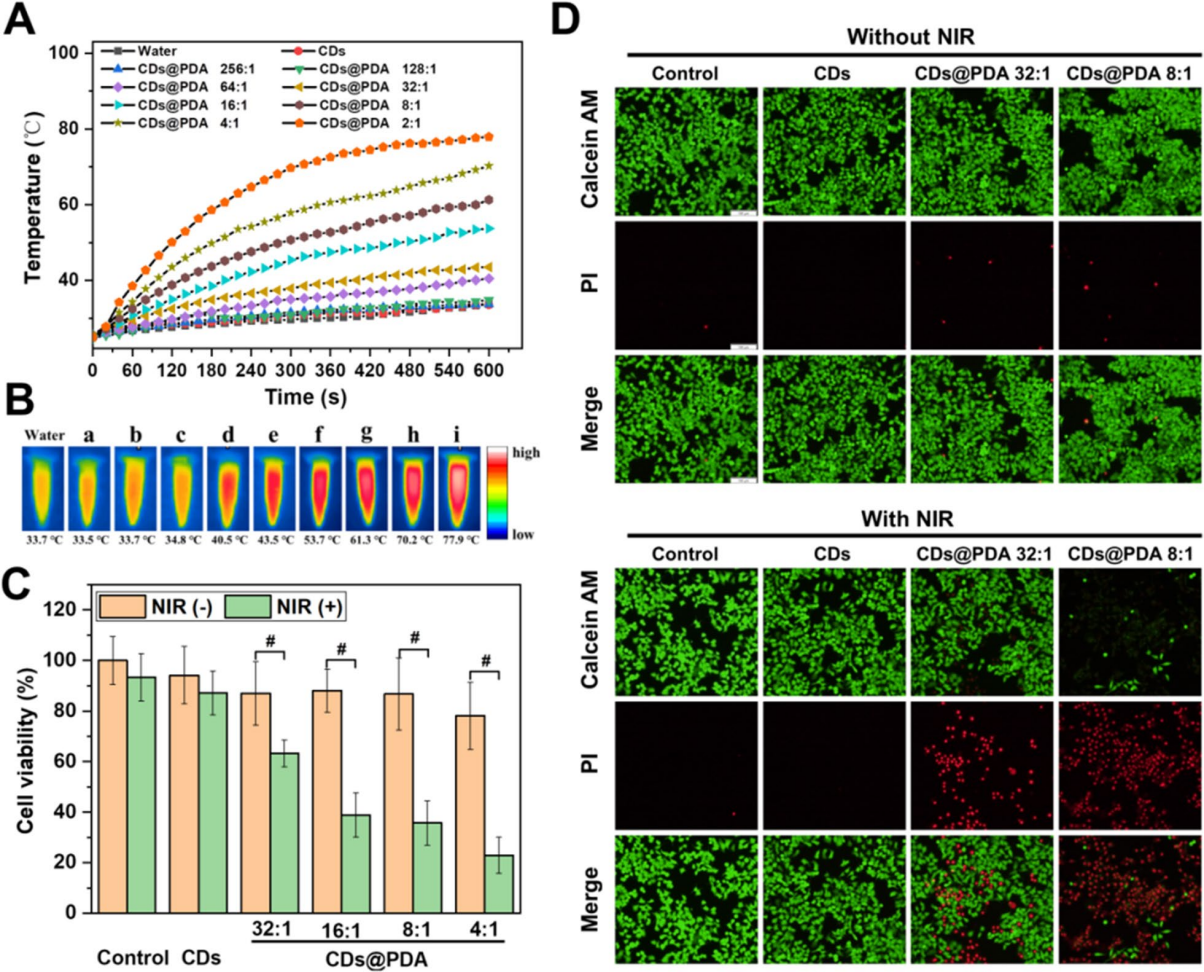
**A** Temperature elevations of CDs@PDA with different CDs/DA ratios upon NIR laser irradiation (808 nm, 1 W/cm^2^, 10 min). **B** IR thermal images of CDs@PDA with different CDs/DA ratios after 10 min irradiation of 808 nm laser (a: CDs, b–i: CDs@PDA with the ratio from 256:1 to 2:1). **C** Cell viabilities of HepG2 cells treated by CDs and CDs@PDA, with or without NIR laser irradiation (808 nm, 2 W/cm^2^, 3 min), ^#^*p* < 0.001. **D** Live-dead staining images of HepG2 cells treated by CDs@PDA with (or without) NIR laser irradiation (808 nm, 2 W/cm^2^, 3 min, scale bar = 100 μm)

Photothermal therapy is one of the most promising applications of PDA, as it has broad band absorption from UV to NIR region and can efficiently convert it into heat to kill cancer cells. In this work, the photothermal effect of CDs@PDA was studied to evaluate the feasibility for photothermal therapy. Firstly, CDs@PDA with different CDs/DA ratios (from 256:1 to 2:1, CDs fixed at 666.7 µg/mL) were irradiated by 808 nm NIR laser at 1 W/cm^2^ for 10 min. Figure 8A exhibited the temperature of each CDs@PDA aqueous dispersion, which was gradually increased with the prolongation of irradiation time. The temperatures of deionized water and CDs aqueous dispersion both slightly increased from 25 to 33 ℃, indicating the photothermal conversion capability of CDs was negligible. However, the photothermal effect of CDs@PDA was heavily dependent on the CDs/DA ratio (exactly the amount of coated PDA), where more PDA led to higher temperature. For CDs@PDA with the ratios of 256:1 and 128:1 (containing 2.6 and 5.2 µg/mL PDA), negligible photothermal capabilities were observed as their temperature variation curves were similar to deionized water. For samples with the CDs/DA ratios of 64:1 and 32:1 (containing 10.4 and 20.8 µg/mL PDA), the temperature increased to about 40 ℃ within 10 min irradiation, indicating a weak photothermal effect occurred. Once the CDs/DA ratios reached to 16:1, 8:1, 4:1 and 2:1 (containing 41.6, 83.3, 166.7 and 333.3 µg/mL PDA), the temperature would rapidly rise under NIR irradiation and finally reached to 54 ℃, 61 ℃, 70 ℃ and 78 ℃. The thermal images also confirmed that CDs@PDA with higher amount of coated PDA exhibited higher photothermal conversion efficiency (Fig. 8B).

Furthermore, the photothermal therapy capability of CDs@PDA on cancer cells was evaluated by MTT assay and live-dead staining. As previously reported, cancer cells would be ablated at 42 °C for 30 min, and the treatment time could be shortened by 20–25 min if the intracellular temperatures exceed 50 °C [20, 40]. Hence, CDs@PDA with the CDs/DA ratios of 32:1, 16:1, 8:1 and 4:1 were selected for photothermal evaluation. CDs and four types of CDs@PDA were incubated with HepG2 cells for 4 h, and then treated with or without NIR laser (2 W/cm^2^, 3 min). As exhibited in Fig. 8C, the cell viabilities of CDs and CDs@PDA were all comparable to the control group, indicating they were good biocompatible to cells. In addition, it seemed the NIR laser irradiation had negligible effect on HepG2 cells of control group and CDs group. However, the viabilities of CDs@PDA groups (CDs/DA ratios of 32:1, 16:1, 8:1 and 4:1) were significantly decreased to 63%, 39%, 36% and 23%, respectively. This demonstrated that CDs@PDA could be easily uptaken by cancer cells and successfully convert NIR energy into heat, which subsequently cause cytotoxic hyperpyrexia to kill cells. Finally, live-dead staining was applied to further confirm the photothermal therapy efficacy of CDs@PDA. HepG2 cells were stained with Calcein-AM and PI, following the treatment of CDs@PDA with (or without) NIR laser irradiation. The dead cells could be identified by PI with red fluorescence, as it could embed in the cellular DNA of dead or apoptosis cell. The live cells could be labeled by Calcein-AM, as it could be cleaved by intracellular esterase to produce calcein emitting intense green fluorescence. As shown in Fig. 8D, HepG2 cells treated by CDs and CDs@PDA without NIR laser irradiation both performed strong green fluorescence and negligible red fluorescence, which were the same as the control group. This meant both CDs and CDs@PDA were nontoxic to cells. By contrast, the cells treated by CDs@PDA combined with NIR exhibited significant red fluorescence, while that treated by CDs and NIR were still almost green fluorescence. This meant that the HepG2 cells were killed by CDs@PDA via photothermal effect. Moreover, CDs@PDA with the CDs/DA ratio of 8:1 killed more cells than that of 32:1, confirming that higher amount of PDA could result in higher photothermal conversion efficiency. Overall, all of the results fully demonstrated that PDA coating endowed CDs with the capability of photothermal conversion. The obtained CDs@PDA were proven to be biocompatible and efficiently kill cancer cells under NIR irradiation. Hence, they could be developed as powerful photothermal agents for cancer therapy.

## Conclusion

In summary, the novel CDs with bright blue photoluminescence were prepared by carbonization of egg yolk. They were approximate sphere in shape with an average size of 4.46 ± 1.17 nm, and exhibited bright blue photoluminescence. The CDs had some charming advantages, including easy mass production, eco-friendly and high quantum yield, which benefit them to be used for multiple application. Firstly, the photoluminescence of CDs could be selectively quenched by Fe^3+^ in a linear manner, and used for Fe^3+^ detection in solution. They could also be uptaken by HepG2 cells and exhibit bright blue photoluminescence for cell imaging, as well as reflect the level of intracellular Fe^3+^. Secondly, the CDs could be coated by PDA on the surface to produce CDs@PDA. We found PDA coating could quench the photoluminescence of CDs via inner filter effect. There was a linear relationship between the degree of quenching and the logarithm of DA concentration (Log C_DA_), which could be potentially developed as dopamine assay. Also, the selectivity experiment indicated the method had a high selectivity for DA over a number of possible interfering species. Finally, the CDs@PDA exhibited excellent photothermal conversion capability, and they could efficiently kill HepG2 cells under NIR laser irradiation for photothermal therapy. Hence, the CDs and CDs@PDA in this work exhibited many excellent advantages, and they could be potentially used for multi-applications, such as Fe^3+^ sensor in solution and in cellular, cell imaging, dopamine assay kit as well as photothermal agents for cancer therapy.

## Supplementary Information

Below is the link to the electronic supplementary material.

Supplementary file1 (DOCX 706 KB)

## Data Availability

The datasets used or analyzed during the current study are available from the corresponding author on reasonable request.

## Abbreviations

CDs: Carbon dots
DA: Dopamine
PDA: Polydopamine
CDs@PDA: Polydopamine-coated CDs
Fe^3+^: Ferric ion
QY: Quantum yield
FRET: Fluorescence resonance energy transfer
NIR: Near-infrared
MR: Magnetic resonance
Tris: Tris(hydroxymethyl)aminomethane
DMEM: Dulbecco’s modified Eagle’s medium
MTT: 3-(4,5-dimethylthiazol-2-yl)-2,5-diphenyltetrazolium bromide
PI: Propidium iodide
FBS: Fetal bovine serum
UV–Vis: Ultraviolet–visible
TEM: Transmission electron microscopic
FTIR: Fourier transform infrared spectroscopic
XPS: X-ray photoelectron spectroscopic
Log C_DA_: Logarithm of dopamine concentration
IFE: Internal filter effect
