# Supplementary material for: Multi-applications of carbon dots and polydopamine-coated carbon dots for Fe3+ detection, bioimaging, dopamine assay and photothermal therapy
Source: Discov Nano. 2023 Mar 2;18(1):30. doi: 10.1186/s11671-023-03809-5 (PMC9981840; doi:10.1186/s11671-023-03809-5)
Supplement: Supplementary file 1 — Supplementary file1 (DOCX 706 KB) [file 11671_2023_3809_MOESM1_ESM.docx]

**Supporting Information**

**Multi-applications of carbon dots and polydopamine coated carbon dots for Fe^3+^ detection, bioimaging, dopamine assay and photothermal therapy**

Jun Chen^1,2†^, Yuting Wang^1†^, Liang Wang^2†^, Mingjie Liu^1^, Linlin Fang^1^, Peng Chu^1^, Chuanzhou Gao^3^, Dapeng Chen^2^, Dongze Ren^1^, Jianbin Zhang^1*^

^1^ Collage of Pharmacy, Dalian Medical University, Dalian 116044, China.

^2^ Laboratory Animal Center, Dalian Medical University, Dalian 116044, China

^3^ Institute of Cancer Stem Cell, Dalian Medical University, Dalian 116044, China

**^†^Jun Chen** and **Yuting Wang** contributed equally to this work.

**^*^ Corresponding authors:**

**Jianbin Zhang,**

College of Pharmacy, Dalian Medical University, 9 West Sect Lvshun South Rd, Dalian 116044, China. Tel: 86-0411-86110420, E-mail: [zhangjb@dmu.edu.cn](mailto:zhangjb@dmu.edu.cn)

**Supporting Information**

**
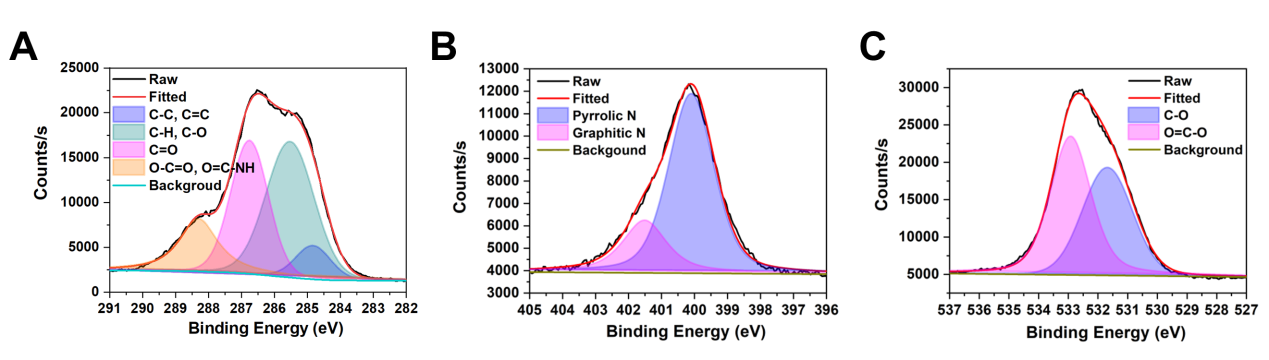
**

**Fig. S1 The high resolution XPS C1s (A), N1s (B) and O1s (C) spectra of CDs.**

**
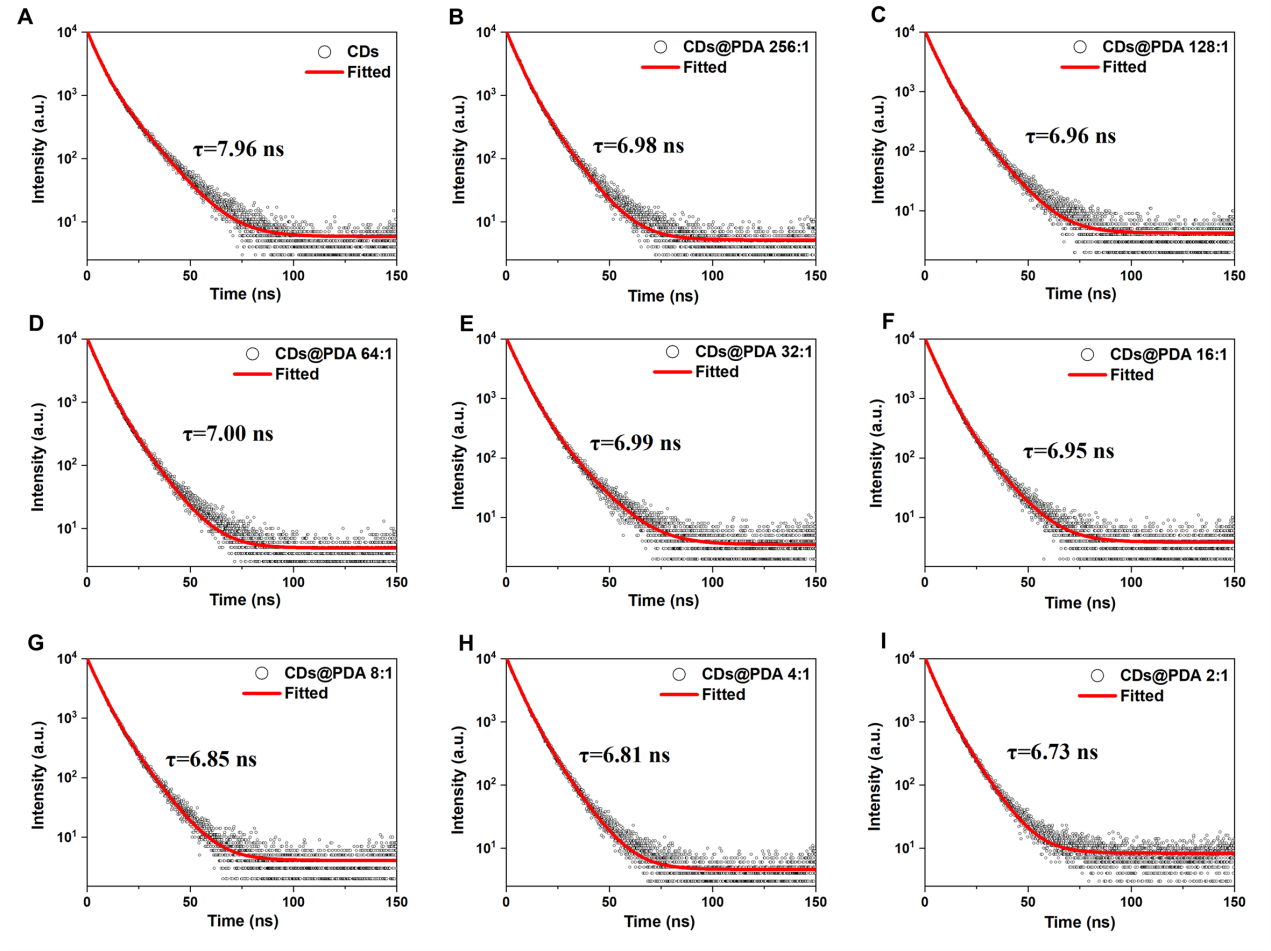
**

**Fig. S2 The lifetime decay curves of CDs and CDs@PDA, with different CDs/DA ratios.**
